# The Doctors

**Published:** 1896-12

**Authors:** 


THE DOCTORS: The following table, compiled by Dr. Fanny Leake, of Austin, Texas, with great pains and care, is interesting, as showing the extent to which the doctors prevail in the United States; and also throws some light upon their relative ability to deal with disease. Those States having less than 500 to each doctor, are embraced in the upper bracket; States having one doctor to 900 or more population, embraced in the lower bracket:

Table showing proportion of population to the doctor—by States—with death-rate in each.

| STATES | DEATH-RATE PER 1000 | POPULATION TO EACH M.D. |
|---|---|---|
| Me. | 15.19 | 567 |
| N. H. | 18.79 | 669 |
| Ver. | 16.32 | 531 |
| Mass. | 20.15 | 555 |
| R. I. | 21.18 | 636 |
| Conn. | 19.52 | 655 |
| N. Y | 20.53 | 538 |
| N. J. | 21.00 | 874 |
| Penn. | 13.98 | 623 |
| Del. | 18.53 | 705 |
| Md. | 17.27 | 520 |
| Va. | 14.03 | 837 |
| W. Va. | 10.85 | 617 |
| N. C. | 11.38 | 1191 |
| S. C. | 13.46 | 1086 |
| Ga. | 11.25 | 909 |
| Fla. | 10.50 | 525 |
| Ala. | 13.81 | 942 |
| Miss. | 11.55 | 944 |
| La. | 14.62 | 766 |
| Tex. | 11.84 | 484 |
| Dist. Col. | 25.85 | 268 |
| Ky. | 12.85 | 599 |
| Tenn. | 13.49 | 574 |
| Ark. | 12.76 | 612 |
| Mo. | 12.16 | 565 |
| Kas. | 8.42 | 645 |
| Ohio. | 13.57 | 848 |
| Ind. | 11.03 | 479 |
| Ill. | 13.88 | 552 |
| Mich | 11.95 | 561 |
| Wis. | 11.06 | 854 |
| Minn. | 11.89 | 826 |

| STATES | DEATH-RATE PER 1000 | POPULATION TO EACH M.D. |
|---|---|---|
| Iowa. | 9.16 | 562 |
| N. Dak. | 9.39 | 901 |
| S. Dak. | 8.22 | 906 |
| Neb. | 7.99 | 663 |
| Mont. | 7.66 | 535 |
| Wy. | 6.82 | 1011 |
| Colo. | 13.23 | 449 |
| Idaho. | 9.14 | 773 |
| Wash. | 7.71 | 537 |
| Ore. | 8.21 | 480 |
| Cal. | 14.65 | 383 |
| Utah. | 10.19 | 818 |
| Ari. | 9.61 | 627 |
| Nev. | 9.48 | 953 |
| States less than 500 to doctor. | | |
| Dist. Col. | 25.85 | 268 |
| Cal. | 14.65 | 383 |
| Colo. | 13.23 | 449 |
| Ore. | 8.21 | 480 |
| Tex | 11.84 | 484 |
| Ohio | 13.57 | 484 |
| Ind. | 11.03 | 479 |
| States over 900 to the doctor. | | |
| N. Dak. | 9.39 | 901 |
| S. Dak. | 8.22 | 906 |
| Ga. | 11.25 | 909 |
| Ala | 13.81 | 942 |
| Miss | 11.55 | 944 |
| Nev | 9.48 | 953 |
| Wy. | 6.82 | 1011 |
| S. C. | 13.46 | 1086 |
| N. C. | 11.38 | 1191 |

DR. TUTWILER calls on the JOURNAL to put in a word for the epileptics, (see his letter). The doctor must certainly have neglected the "Red Back; for—in April and May and June a large share of our space was devoted to the subject—papers by Dr. Peterson and by Dr. White and others on the subject being published; to say nothing of editorial comment, until we began to fear the subject was becoming tiresome. In the last issue—November—there was a mention of the subject; it was stated that the committee appointed at Fort Worth to memorialize the legislature in accordance with resolutions passed at that meeting of the Association were at work, and by the time the legislature assembles, will be ready with their memorial—petitioning the State to make provision for the epileptics.

The JOURNAL, however—ever ready to espouse the cause of the unfortunates—again assures its readers of its active interest in the subject, and that we will, by word and pen, do what lies in our power to promote the undertaking to arouse State interest in this neglected class.

